# Telehealth-based exercise in amyotrophic lateral sclerosis

**DOI:** 10.3389/fneur.2023.1238916

**Published:** 2023-07-26

**Authors:** Virginia Kudritzki, Ileana M. Howard

**Affiliations:** ^1^Rehabilitation Care Services, VA Puget Sound Healthcare System, Seattle, WA, United States; ^2^Department of Rehabilitation Medicine, University of Washington, Seattle, WA, United States

**Keywords:** amyotrophic lateral sclerosis, telehealth, telemedicine, rehabilitation, exercise

## Abstract

The Veterans Health Administration (VHA) has served as a leader in the implementation of telerehabilitation technologies and continues to expand utilization of non-traditional patient encounters to better serve a geographically and demographically diverse population. Amyotrophic Lateral Sclerosis (ALS) is a progressive neurodegenerative disease impacting Veterans at a higher rate than the civilian population and associated with high levels of disability and limited access to subspecialized care. There is growing evidence supporting exercise-based interventions as an independent or adjunctive treatment to maintain or restore function for this patient population; many of these interventions can be delivered remotely by telehealth. The recent advancements in disease-modifying therapies for neuromuscular disorders will likely increase the importance of rehabilitation interventions to maximize functional outcomes. Here, we review the evidence for specific exercise interventions in ALS and the evidence for telehealth-based exercise in neuromuscular disorders. We then use this existing literature to propose a framework for telehealth delivery of these treatments, including feasible exercise interventions and remote outcome measures, recommended peripheral devices, and an example of a current remote group exercise program offered through VHA.

## Introduction

Amyotrophic Lateral Sclerosis (ALS) is a progressive neurodegenerative disease with an estimated lifetime prevalence of one in 400 adults in the United States (1). Veterans experience an even greater risk of developing ALS, with nearly twice the rate of their civilian counterparts (2). As a result, the Veterans Healthcare Administration made ALS a 100% presumptively service-connected condition in 2008 (3).

The standard of care for ALS consists of multidisciplinary care visits quarterly (4), however, interval therapy follow-ups are frequently needed. With over 50% of persons with ALS in the United States living >50 miles away from the nearest ALS specialty center (5), there is a significant barrier for persons with ALS to access a therapist for the multiple visits generally required to follow through a treatment plan.

Physical therapy (PT) is an important component of care for persons with ALS. The role of the physical therapist in the ALS specialty program is to support prevention of secondary complications, restoration of strength and function when possible, and provision and training in strategies and durable medical equipment to compensate for lost function. Therapist expertise in ALS is needed to avoid exacerbation of symptoms such as fatigue, weakness, dyspnea, or injury related to falls. Additionally, an understanding of the typical progression of ALS allows the therapist to anticipate disease progression to ensure appropriate durable medical equipment (DME) provision, which will meet both the current and future needs of the patient-saving time and cost to both patient and the healthcare system by avoiding redundant prescriptions of equipment as the degree of disability advances. Unfortunately, community-based outpatient physical therapists may have limited experience in providing care to patients with ALS.

Advances in disease-modifying therapies have the potential to slow progression and extend life expectancies in ALS, thereby shifting even more focus on the therapist’s role in overseeing a rehabilitative exercise program to enhance functional outcomes. With this change, the ability to access PT for interval care between quarterly visits will become more critical for the person with ALS to ensure adequate supervision and progression toward the goals of treatment. Unsupervised home exercise programs for ALS have been studied, and while patients benefit with improved function, a high drop-out rate has been reported (6).

Use of telehealth for provision of specialized services for persons with ALS has been found to be efficient and economical means to deliver evidence-based interdisciplinary care (7), however, best practices for delivery of interval PT rehabilitative interventions for this population remain undefined. In this narrative review, we will provide an overview of the literature on exercise in ALS, which will be followed by discussion on implementation of exercise modalities to a virtual care setting based upon existing evidence in neuromuscular disease. Remote outcome measures, exercise interventions which can be delivered by telehealth, and peripheral devices used to enhance the collection of remote assessments on the patient’s end will be explored.

## Specific exercises and adaptability to telehealth

### Range of motion exercises

Throughout all stages of ALS, maintenance of range of motion is important to improve mobility, self-care, posture, and wheelchair seating and reduce pain, spasticity, and risk of wounds. Range of motion exercises are considered foundational in ALS care due to the anticipated loss of strength and function. For this reason, range of motion has not been studied as a stand-alone exercise intervention but is often included as part of intervention and control arms for exercise-based studies in ALS.

Guiding patients and their caregivers through a passive or active range of motion program via telehealth is feasible and can be augmented with printed information or videos. Patients and caregivers benefit from detailed instructions for stretching technique and dosing, as well as caregiver hand position, location in reference to the patient, and body mechanics may also be taught, but may be most effective if the therapist can demonstrate on another individual. Successful telehealth-based stretching programs have been described in the orthopedic literature (8).

### Aerobic exercises

Persons with ALS have reduced aerobic capacity related to loss of lean muscle mass and this may be compounded by physical deconditioning (9). Fatigue, declining function, and decreased activity tolerance are the most commonly reported secondary symptoms in the ALS population and may indicate reduced efficiency and aerobic capacity. There is conflicting evidence regarding the effect of aerobic exercise as compared to standard neurorehabilitation interventions in persons with ALS in regard to outcomes including function, quality of life, aerobic capacity, strength, respiratory function, fatigue, and pain (10, 11). High intensity exercises are not advised in ALS due to literature from animal-based studies suggesting worsening rate of progression related to overexertion (12).

Aerobic exercise may be the most amenable to remote service delivery or supervision by telehealth as it can generally be performed and monitored with inexpensive or no equipment. Stationary peddlers or walking programs are commonly used in this population. The intensity of aerobic exercise may be monitored by heart rate or subjective ratings of patient tolerance [e.g., Borg or rate of perceived exertion (RPE) scale]. Braga et al. (13) reported a successful feasibility study in 10 patients with ALS involving a telerehabilitation-based walking program using a treadmill or outdoors walking for persons with ALS, with remote vital signs monitoring (heart rate and pulse oximetry) to ensure safety.

### Resistance exercises

Resistance exercises have been frequently studied in ALS due to weakness being usually the primary impairment associated with this disease. However, several precautions are commonly followed when prescribing exercise to persons with ALS to avoid injury, including avoidance of strengthening exercises to muscles with less than anti-gravity strength (14), and avoidance of eccentric strengthening exercises (15). Moderate intensity for strengthening exercises is gaged by the ability of the patient to complete a high number of repetitions with good form (16). There are mixed results of studies evaluating the effect of resistance exercise as an isolated intervention as compared to “usual care” on functional outcome measures in ALS (10, 11).

Resistance exercise also translates well to remote service delivery, given that equipment is generally inexpensive and widely available. A trial of telehealth-supervised resistance exercise has been described in a population of young adults with cystic fibrosis (17), but not as an isolated intervention in persons with ALS.

### Combined interventions

Combined exercise interventions usually describe programs including both aerobic and resistance exercises. Research on combined interventions in persons with ALS show more promising results than aerobic or resistance exercise alone impacting not only limb strength and aerobic capacity, but also positive effects on function, quality of life, and reduced pain (11, 18). Based upon data from a review of 10 randomized control trials, Ortega-Hombradros (19) concluded that combined resistance and aerobic exercise programs should be completed at a moderate intensity, 2x/week to maximize benefits and reduce risk of worsening fatigue. Effective telehealth multimodal telerehabilitation exercise programs have been described in the Parkinson’s (20) and Duchenne muscular dystrophy (21) populations.

### Respiratory exercises

Dyspnea, orthopnea, hypoventilation, and poor airway clearance are common indications of respiratory dysfunction resulting from neuromuscular weakness in ALS. Respiratory exercises may include lung volume recruitment (breath stacking, glossopharyngeal breathing), muscle strengthening (inspiratory/expiratory muscle training), and airway clearance techniques [manually assisted cough (MAC), huff or squeeze coughing]. Clinical trials of respiratory muscle strengthening exercises in ALS have yet to show significant impact on meaningful endpoints, such as survival, hospitalization, or initiation of invasive mechanical ventilation. In a recently published systematic review (22), respiratory training was not shown to have a significant impact on ALS Functional Rating Scale-Revised (ALSFRS-R) scores or forced vital capacity (FVC). That said, techniques such as lung volume recruitment and airway clearance techniques may be lifesaving in the case of a medical emergency for a person with neuromuscular respiratory weakness.

Respiratory exercises require very little equipment and are easily adaptable to a telehealth setting. Telehealth video instructions for respiratory exercises were well-received by families of young men with Duchenne Muscular dystrophy during the COVID-19 pandemic (23).

### Balance exercises

Persons with ALS are at a high risk of falls and serious fall-related injury (24), therefore, effective balance and fall prevention interventions are of great potential value in this population. Decreased lower limb strength in ALS correlates to increased risk of falls (25), however, specific exercises interventions to mitigate the risk of falls in isolation has not been studied in this population.

Telehealth evaluations for fall prevention are advantageous because of the ability to assess the patient in their home environment, which allows the clinician to identify environmental risk factors for falls as well as assist in appropriate DME prescription for fall prevention. Telehealth-based balance interventions have been found to be feasible and effective in stroke survivors (26) and community-dwelling elderly with balance impairments (27, 28).

## Implementation of telerehabilitation-based exercise for ALS

The Veterans Health Administration (VHA) is well poised for innovation and delivery of specialized therapy services remotely through telehealth. Common barriers impacting the private sector, such as insurance coverages and interstate licensing for telehealth, do not exist in VHA (29). In the sections to follow, pragmatic considerations for further development of programs to serve Veterans with ALS will be explored.

### Asynchronous telerehabilitation

Asynchronous care through emails, text messages, mobile device applications, or through transfer of video files provides the most flexibility for both the clinician and patient and has been used to support physical therapy exercise interventions (30). This flexibility of asynchronous exercise videos may lend to higher utilization as compared to live workshops, as described in one study performed in a population of patients with Duchenne muscular dystrophy and caregivers—the live session reported 16 total participants, whereas the recorded workshop received 132 views within 1 month (31). One limitation to this approach, however, is the inability to resolve concerns, adapt exercises, or obtain additional information in real time when needed.

Automated exercise reminders or check-ins, such as the VHA’s mobile application “Annie,” can further support adherence to the prescribed exercise program or elicit outcomes from Veterans for safety and tolerability monitoring (32). App-based asynchronous combined exercise programs have demonstrated improved functional outcomes for persons with multiple sclerosis (33). There is evidence which demonstrates the effectiveness of asynchronous interventions to support adherence to exercise programs in ALS and ensure safety through monitoring of heart rate and pulse oximetry during exercise sessions (13).

### Synchronous telerehabilitation

Both telephone and clinical video telehealth have been described for synchronous care delivery for ALS. Telephone calls can be used to assess adherence and address any concerns regarding an individualized the exercise program (34). Video telehealth allows interactive and real-time streaming of individual or group therapy interventions to the patient, which may facilitate necessary adaptations to the prescribed exercise program based upon the patients function and tolerance of treatment. VHA served as early adopters of telerehabilitation specifically for physical therapy to facilitate access to rural-dwelling patients (35). One study examining telerehabilitation for persons with ALS during the COVID-19 pandemic confirmed that the highest utilization for synchronous telerehabilitation services was for physical therapy (53.7% of all visits) and that patient satisfaction with this modality was high (>94% “very good” or “excellent”) (36).

### Outcome measurements

Baseline and interval assessments ensure progress is being made toward functional improvements during treatment. In addition, these measures ensure adequate tolerance and safety to participate in exercise-based interventions. Many commonly performed assessments, both objective and subjective measures, have been adapted to use with telehealth. Examples of commonly used measures are included in Table 1.

**Table 1:** Sample remote outcome measures for telerehabilitation in ALS.

Objective measures	Subjective measures
Two minute step test	ALS-FRS
Five times sit to stand	Fatigue severity scale
Pulse oximetry	ALS-specific quality of life
Spirometry	Pain visual analog scale
Heart rate	Rate of perceived exertion (Borg)

### Peripheral devices and technology

Peripheral devices may support teletherapy services by supplementing the physical examination with additional objective data. Technology innovations may assist clinicians in processing data collected from peripheral devices or may enhance the exercise interventions to improve user engagement (37). Although not an exhaustive list, representative examples of peripheral devices and technology which may be applied to the care of persons with ALS are presented in this section.

### Wearable technology

Pulse oximeters can support safe exercise for persons with ALS by monitoring oxygen saturation and pulse before, during, and after a remote exercise session. Patients with ALS who have no co-morbid lung disease should be instructed in the oximetry feedback protocol described by Bach et al. (38), which prescribes use of assisted cough device and non-invasive ventilatory support for oxygen saturation below 95%.

Stand-alone heart rate monitors can be used to assess the intensity of exercise to ensure safety and therapeutic dose of exercise for the participant before and during exercise sessions. Target heart rate of 50–70% maximum heart rate is generally advised for moderate intensity exercise in ALS (39).

Wearable sensors present opportunities for additional objective data at both the impairment and the functional level. Electrogoniometers may be worn to quantify range of motion and/or detect changes in available range of motion over time. Accelerometers are commonly used for fall detection purposes and can also be used for remote monitoring of physical activity levels (40, 41). In addition, wearable sensors can provide information about gait characteristics (42).

### Spirometers

Home spirometry is a convenient means of monitoring respiratory function for patients enrolled in virtual exercise programs and is more informative than pulse oximetry for monitoring neuromuscular respiratory failure (43). In addition to monitoring effects of an exercise program, earlier detection of respiratory compromise through more frequent monitoring at home with or without clinician supervision may also lead to earlier initiation of supportive interventions such as non-invasive ventilatory support (44).

### Exergaming

Single camera video game peripherals have been used to measure reachable workspace related to upper body function in ALS (45). Exergames, or video games utilized for therapeutic rehabilitation purposes to improve adherence, must be adaptable for persons with disability to accommodate for progressive change in physical function. When this is taken into account, patients with neuromuscular disease and low functional abilities report satisfaction and enjoyment from engaging in this treatment (46). Virtual reality has been described as an adjunct technology to assist with upper body exercises in persons with ALS by customizing and gamifying tasks within the reachable workspace of the individual (47).

### Artificial intelligence

Artificial Intelligence and machine learning will be critical components to process data elements derived from wearable technology to filter results and alert the medical team to notable changes in the patient’s medical condition. One recent study described the use of machine learning to predict ALS-FRS scores based upon electrogoniometer and speech recording results (48). Digital health technology for ALS must be vetted by subject matter experts in the field to ensure accurate interpretation.

## Program example: ALS HOPE

The ALS Holistic OutPatient Exercise (HOPE) program is a synchronous video telehealth-based exercise group designed for Veterans with ALS receiving care at the VA Puget Sound. Implemented in December 2022, this group is directed toward individuals with independent mobility, greater than anti-gravity strength in at least one limb, and ALS-FRS >32. Veterans participate in group exercise incorporating range of motion, strengthening, balance, and respiratory exercises, adapted for their individual level of function, under the supervision of a specialized ALS team physical therapist and physical therapy assistant. Outcomes assessed include all measures listed in Table 1 and are assessed at baseline and repeated at 6, 12, and 24 weeks. The group meets twice per week for 1 h per session and enrollment occurs on an ongoing basis. Follow-up assessments were conducted for practice improvement. This operational analysis was reviewed jointly by the Human Research Protection Program and Quality, Safety & Value service line at the VA Puget Sound Health Care System and determined to not constitute human subjects research.

During the 6-month pilot phase of this program, seven Veterans enrolled in the exercise group on a rolling basis. One Veteran discontinued after moving into a facility which offered an exercise program, the remainder continued participation with an average of 25 sessions (range 10–31). Average travel saved per participant for each session was 42 miles. No serious adverse events were reported in this timeframe. Participants report high levels of satisfaction with the program, citing ease of use, experiencing different points of view of a shared experience, and the ongoing connection to the ALS team between quarterly visits.

This program serves as an example of an intervention to increase access and support with the aim of reducing symptoms and maximizing function for Veterans with ALS, regardless of their geographic location.

## Limitations and future directions

There is still ambiguity of the safety and efficacy of exercise for persons with ALS. Heterogeneity of participants and studies, small sample sizes, and presence of bias have created inconsistent results and unclear guidance on how to implement exercise into the healthcare plan.

Group telehealth exercise interventions may be an efficient way to improve access and efficiency of care, not only for PT, but also for all other subspecialized ancillary clinical services needed for optimal ALS care. During the COVID-19 pandemic, the VHA’s Gerofit group exercise program rapidly and successfully adapted protocols for physical function assessments and group exercise treatments to a telehealth, accommodating up to 24 geriatric patients in a virtual session (49). A similar transformation for other specialized services in ALS, such as assistive technology, or driver’s rehab, for example, could have significant impact on the quality of life for persons with ALS.

## Discussion

Most exercises described for persons with ALS fall into one of the following categories: range of motion (stretching), strengthening, or combined exercises. The strongest evidence supports combined exercise interventions, which have demonstrated improved outcomes, including increased aerobic capacity, strength, function, and quality of life for affected persons. Most of these interventions involve widely available and low-cost equipment, making them relatively simple to translate to the virtual care setting. Supervision of an exercise program by a physical therapist with knowledge of ALS is critical to ensure appropriateness of exercise modalities, necessary adaptations for the patient’s functional level, and ensure safety, tolerability, and efficacy. Given the geographic limitations on specialty ALS centers, telehealth-based exercise is an attractive option for care service delivery in this population.

Innovative approaches to telerehabilitation, including asynchronous app-based and synchronous clinical video telehealth have been promoted by VHA and were accelerated outside of the VHA by the COVID-19 public health emergency. The ALS HOPE group exercise program is an example of a promising model of care, which could potentially improve the efficiency of delivery of care by providing interval subspecialized therapy beyond the usual quarterly multidisciplinary team visit. Further work is needed to confirm the effectiveness of telerehabilitation for persons with ALS and to compare the efficacy of telerehabilitation group-based interventions to individualized treatment in this population.

Even with the modest evidence of benefit for exercise for persons with ALS, the low risk and low cost associated with this treatment favors routine implementation of a combined exercise program for persons at early stages of disease.

## Data availability statement

The original contributions presented in the study are included in the article/supplementary material, further inquiries can be directed to the corresponding author.

## Author contributions

IH and VK decided the idea and structure, and contributed to research, writing, revision, and reading. All authors contributed to the article and approved the submitted version.

## Funding

The work reported here was supported by the Department of Veterans Affairs, Veterans Health Administration, Office of Rural Health, Veterans Rural Health Resource Center-Gainesville, ALS Comprehensive Care Supported by CVT in the Home (NOMAD ID PROJFY-08810) under national lead, Amy Kunce, MS, BSRS. Funding for publication was provided by The ALS Association.

## Conflict of interest

The authors declare that the research was conducted in the absence of any commercial or financial relationships that could be construed as a potential conflict of interest.

## Publisher’s note

All claims expressed in this article are solely those of the authors and do not necessarily represent those of their affiliated organizations, or those of the publisher, the editors and the reviewers. Any product that may be evaluated in this article, or claim that may be made by its manufacturer, is not guaranteed or endorsed by the publisher.
